# Smooth muscle NOS, colocalized with caveolin-1, modulates contraction in mouse small intestine

**DOI:** 10.1111/j.1582-4934.2008.00335.x

**Published:** 2008-04-08

**Authors:** Ahmed F El-Yazbi, Woo Jung Cho, Jonathan Cena, Richard Schulz, Edwin E Daniel

**Affiliations:** a Department of Pharmacology, University of AlbertaEdmonton, Alberta, Canada; b Department of Pediatrics, University of AlbertaEdmonton, Alberta, Canada

**Keywords:** nitric oxide synthase, caveolin-1, smooth muscle contraction, mouse small intestine

## Abstract

Neuronal nitric oxide synthase (nNOS) in myenteric neurons is activated during peristalsis to produce nitric oxide which relaxes intestinal smooth muscle. A putative nNOS is also found in the membrane of intestinal smooth muscle cells in mouse and dog. In this study we studied the possible functions of this nNOS expressed in mouse small intestinal smooth muscle colocalized with caveolin-1(Cav-1). Cav-1 knockout mice lacked nNOS in smooth muscle and provided control tissues. 60 mM KCl was used to increase intracellular [Ca^2+^] through L-type Ca^2+^ channel opening and stimulate smooth muscle NOS activity in intestinal tissue segments. An additional contractile response to LNNA (100 μM, NOS inhibitor) was observed in KCl-contracted tissues from control mice and was almost absent in tissues from Cav-1 knockout mice. Disruption of caveolae with 40 mM methyl-β cyclodextrin in tissues from control mice led to the loss of Cav-1 and nNOS immunoreactivity from smooth muscle as shown by immunohistochemistry and a reduction in the response of these tissues to *N*-ω-nitro-L-arginine (LNNA). Reconstitution of membrane cholesterol using water soluble cholesterol in the depleted segments restored the immunoreactivity and the response to LNNA added after KCl. Nicardipine (1 μM) blocked the responses to KCl and LNNA confirming the role of L-type Ca^2+^ channels. ODQ (1 μM, soluble guanylate cyclase inhibitor) had the same effect as inhibition of NOS following KCl. We conclude that the activation of nNOS, localized in smooth muscle caveolae, by calcium entering through L-type calcium channels triggers nitric oxide production which modulates muscle contraction by a cGMP-dependent mechanism.

## Introduction

Nitric oxide is a key regulator of multiple biological processes in different organs and systems in the body. It is synthesized by the reaction of L-arginine and oxygen catalysed by a group of enzymes called nitric oxide synthases (NOS) to produce nitric oxide and citrulline. At least three distinct isoforms of NOS exist in mammalian cells: endothelial NOS (eNOS), neuronal NOS (nNOS), and inducible NOS (iNOS) [1]. Ca^2+^-dependent NOS activity was first localized in neurons by Bredt *et al.*[2] where it releases nitric oxide to act as a neurotransmitter or retrograde messenger, and was later named neuronal NOS. However, following its identification in neurons, further examination of nNOS distribution showed that it existed in several other tissues including skeletal muscle [3], vascular smooth muscle [4] and cell membrane of intestinal smooth muscle in dog [5] and mouse [6].

The earliest examination of the function of nNOS expressed outside nerve cells was in skeletal muscle tissue [7]. There, the nitric oxide produced was shown to modulate the contractile activity of skeletal muscle through the activation of soluble guanylate cyclase. In vascular smooth muscle, nitric oxide produced by nNOS was shown to be responsible for reduced vascular contractility in hypoxia [8]. In dog lower oesophageal sphincter, smooth muscle nNOS activity was shown to be important in the modulation of the generated tone through the regulation of potassium channel activity [5].

Similar to eNOS, nNOS activity was shown to be regulated *in vitro* by its interaction with caveolin-1 (Cav-1) [9]. Cav-1 is a member of a family of 21–24 kD integral membrane proteins that insert into the inner leaflet of plasma membrane and form hetero-oligomers, responsible for the formation of the characteristic flask-shaped caveolae [10]. Caveolae act as membrane organizing centres that recruit lipids and proteins, through specialized motifs in caveolins, to participate in intracellular trafficking and signal transduction [11]. Cav-1 and 3 bind to and regulate the activity of a number of signalling molecules including, in addition to eNOS and nNOS, Src family kinases, heterotrimeric G proteins, H Ras, protein kinase C and adenylyl cyclase [reviewed in 12]. Cav-1 was shown to inhibit nNOS activity by interfering with calcium/calmod-ulin binding [9, 13]. Moreover, examination of the *in vivo* distribution of nNOS and caveolin proteins showed that they were colocalized in skeletal muscle plasma membrane [9] and cell membranes of vascular smooth muscle [14], canine lower esophageal sphincter muscle [15] mouse small intestinal smooth muscle and interstitial cells of Cajal [16].

Cav-1 knockout mice were shown to lack morphologically identifiable caveolae in tissues expressing Cav-1. They also showed a number of abnormalities including defects in caveolar endocytosis, lung hypercellularity, decreased vascular tone and atrophic fat pads [17]. We recently showed that they have a defective relaxation in response to nitric oxide released by the stimulation of enteric neurons [18, 19] and have an altered response to β-adrenoceptor stimulation [20]. We also showed that these mice lack a putative nNOS variant expressed in intestinal smooth muscle cells [18]. Thus, in the present study, we compared responses to increased intracellular Ca^2+^ concentration in Cav-1 knockout and wild-type mice to address the possible function of this putative nNOS expressed in small intestinal smooth muscle.

## Materials and methods

All animal experiments were conducted according to a laboratory animal protocol approved by the University of Alberta Animal Policy and Welfare Committee.

### Functional experiments

#### Tissue preparation

Male 6–8 week-old BALB/c, Cav-1 knockout [(cav<tm 1 M ls>/J) (cav1^−/−^), and genetic control [(B6 129 SF2/J) (cav1^+/+^)] mice (Jackson Laboratory, Bar Harbor, Maine) were killed by cervical dislocation. After opening of the abdominal wall, the digestive tract, starting from the stomach to the rectum, was removed from the mouse and immediately placed into a beaker of Krebs-Ringer solution at room temperature (21–22°C) containing (in mM): 115.5 NaCl, 21.9 NaHCO_3_, 11.1 D-glucose, 4.6 KCL 1.16 MgSO_4_, 1.16 NaHPO_4_ and 2.5 CaCl_2_. In a dissection dish filled with Krebs-Ringer solution and continuously bubbled with carbogen (95% O_2_ and 5% CO_2_), small intestinal tissue (jejunum) was isolated and cut into approximately 0.5 cm segments. The tissue segments were mounted to record the circular muscle contractile activity as described previously [21]. Briefly, the open side of a thin metal triangle was slid through the lumen of the tissue segment. The triangle was then hooked together so that its base passed axially through the lumen of the tissue segment. A stainless steel rod attached to the bottom of an electrode holder was also inserted to pass axially into the lumen of the tissue segment parallel to and beneath the base of the metal triangle. In such an arrangement, contraction of the tissue segment around the base of the triangle and metal rod brings them closer. Silk suture thread, attached to the apex of the triangle opposite to the tissue, was tied to a force displacement transducer (Grass FT-03). Two thin platinum rods, situated on both side of and parallel to the tissue, were used for the electrical stimulation of the tissue. The muscle preparations were placed in muscle baths filled with Krebs-Ringer solution, continuously bubbled throughout the experiment with carbogen, and maintained at a temperature of 37°C. The tension on the tissue was adjusted to 0.5 g tension and varied slightly to get the maximum phasic activity. Tissue contractile activities were recorded on a Grass Model 7D Polygraph. At the end of the experiments, tissues were washed and incubated in calcium-free Krebs-Ringer solution with 1 mM ethylene glycol tetra-acetic acid (EGTA) to bring them down to the basal passive tension. The tension spontaneously produced by the tissue was measured to the passive tension level in Ca^2+^-free Krebs solution.

#### Experimental protocols

Neuronal activity in intact tissue segments was blocked by 1 μM tetrodotoxin (TTX) and ω-conotoxin GVIA (1 μM). The blockade was ascertained by examining the response to electric field stimulation after TTX. Tissues were equilibrated for 25 min. After the equilibration period, tissues were contracted using 60 mM KCl and the contractions were allowed to plateau. In some experiments, cyclopiazonic acid (1 μM) or carbachol (CCh, 10 μM) were added in place of KCl. Following the tissue plateau or after 5 min. following the addition of any of the previous drugs, the tissues were treated with *N-*ω-nitro-L-arginine (LNNA, 100 μM), oxadiazoloquinazolinone (ODQ, 1 μM), apamin (1 μM) or iberiotoxin (Ibtx, 0.1 μM). In some of the KCl and CCh experiments, the tissues were pre-treated with nicardipine (1 μM).

In some experiments membrane cholesterol was depleted by incubating the control mouse tissue for 1 hr at 37°C in Krebs-Ringer solution containing 40 mM methylβ-cyclodextrin (Me-β-CDX) and continuously bubbled with carbogen. In some of these tissues 2.54 mM water soluble cholesterol (WSC) in Krebs-Ringer was incubated for 1 hr at 37°C to replete the membrane cholesterol. KCL and LNNA were added to cholesterol depleted tissues and tissues treated with Me-β-CDX and WSC.

#### Data analysis and statistics

The increase in tissue contractile tone was measured 5 min. following the addition of LNNA at which time the contraction of the tissue had plateaued. The increase in the tone of contraction following LNNA addition was normalized to the active tissue tone prior to the addition of KCl. The active tissue tone was measured as the difference between the lowest level of contraction the tissue attains during phasic activity and the basal passive tension brought about by calcium-free Krebs with 1 mM EGTA at the end of the experiment. The absolute increase in tension showed the same trends and same statistically significant differences as the normalized values. The results are indicated as mean ± standard error and *n* represents the number of animals whose tissues were used for the particular experiment. Statistical comparisons were done using anova followed by Bonferroni *post hoc* test or unpaired t-test whichever appropriate. A *P*-value <0.05 was considered significant.

### Immunohistochemistry

#### Tissue preparation

The gastrointestinal tract was obtained from Cav1^+/+^ and Cav1^−/−^ mice and put into ice-cold oxygenated (95% O_2_ and 5% CO_2_) Krebs-Ringer solution. The jejuni of both Cav-1^+/+^ and Cav-1^−/−^ were prepared for both cryosection and whole mount preparation. In addition, BALB/c jejuni were obtained after muscle bath experiment (treatment with Me-β-CDX and WSC) and prepared for cryosection.

#### Cryosection

The jejuni were opened along the mesenteric border, pinned on a petri-dish lined with Sygard silicon rubber, and fixed with 4% paraformaldehyde in 0.1 M sodium phosphate buffer for 4 hrs. The fixed jejuni were rinsed with 0.1 M phosphate buffer eight times every 1 hr and cryoprotected with 30% sucrose in 0.1 M phosphate buffer overnight at 4°C. The cryoprotected jejuni were trimmed and put into peel-a-way disposable embedding moulds filled with Tissue-Tek® O.C.T. compound in order to obtain optimal sections. The embedding molds were frozen for 1 hr at -28°C, peeled, and trimmed for cryosection. 6 μm cryosections of both Cav-1^+/+^ and Cav-1^−/−^ were obtained by a cryostat and attached to a glass slide coated with 1.5% 3-aminopropyltriethoxysilane in acetone. The cryosections were dried for 30 min. at room temperature.

#### Whole mount preparation

The jejuni of both Cav-1^+/+^ and Cav-1^−/−^ were opened along the mesenteric border, stretched to about 200% of original size, pinned on a petri-dish lined with Sygard silicon rubber and fixed with 4% paraformaldehyde in 0.1 M phosphate buffer for 4 hrs at room temperature. The fixed jejuni were rinsed with 0.1 M phosphate buffer eight times every 1 hr, cleared with dimethylsulfoxide three times every 10 min. and re-rinsed in phosphate-buffered saline three times every 15 min. at room temperature. The jejuni were trimmed to 1.0 × 1.5 cm^2^ (circular muscle layer × longitudinal muscle layer). In trimmed jejuni muscular layers (circular and longitudinal muscle layer) were separated from the mucosa and submucosa layers. To obtain myenteric plexus attached to longitudinal muscle layer, muscle fibres of the circular muscle layer were peeled away by tweezers under a dissection microscope.

#### Immunolabeling of cryosesctions

The dried cryosections were rinsed with 0.3% Triton-X 100 in phosphate-buffered saline twice every 5 min. to facilitate penetration of primary antibody and remove O.C.T. compound, followed by rinsing with phosphate-buffered saline once for 5 min. To reduce non-specific binding to proteins, 10% normal donkey serum in phosphate buffer saline was applied on the cryosection for 30 min. at room temperature. Primary antibodies (mouse anti-Cav-1 and a guinea pig anti-nNOS COOH- epitope, which recognizes the nerve nNOS [22] and the putative smooth muscle nNOS [15] or a rabbit anti-nNOS COOH terminal epitope, which was used to stain nNOS expressed in vascular smooth muscle [23], were mixed together and incubated for 16–17 hrs. The cryosections were rinsed with 0.3% Triton-X 100 in phosphate-buffered saline twice every 5 min., followed by a wash with phosphate-buffered saline once for 5 min. Two different secondary antibodies (Cy3-conjugated donkey antimouse IgG and fluorescein isothiocyanate (FITC)-conjugated donkey anti-guinea pig IgG) were mixed together and incubated for 1.5 hrs. The cryosections were rinsed with 0.3% Triton-X 100 in phosphate-buffered saline twice every 5 min., followed by a wash with phosphate-buffered saline once for 5 min. Incubation procedures were performed at room temperature.

#### Immunolabelling for whole mount preparation

Th myenteric plexus attached to longitudinal muscle preparations were put into a 24-tissue well plate and rinsed with 0.5% Triton-X 100 in phosphate-buffered saline for 1 hr. 10% normal donkey serum in 0.5% Triton-X 100 in phosphate-buffered saline was applied on the preparations for 1 hr at room temperature. Primary antibodies (mouse anti-HuC/D, which labels neuron cell bodies, and guinea pig anti-nNOS COOH- epitope were mixed together and incubated for 64–65 hrs. The preparations were rinsed with 0.5% Triton-X 100 in phosphate-buffered saline three times every 15 min. Two different secondary antibodies (Cy3-conjugated donkey anti-mouse IgG, and FITC-conjugated donkey anti-guinea pig IgG) were mixed together and incubated for 3 hrs. The preparations were rinsed with 0.5% Triton-X 100 in phosphate-buffered saline twice every 15 min. followed by a wash with phosphate-buffered saline once for 15 min. Incubation procedures were performed at 4°C.

All antibodies and normal sera used are summarized in Table 1. During the incubation with primary antibodies, 1% normal serum of total incubation volume that was raised in the host species of the secondary IgG was added. To determine specificity of immunolabelling, primary antibody was omitted, or when the antigen was available, it was used to saturate the primary antibody.

**Table 1:** Antibodies and normal serum.

Antibody	Host	Dilution	Peptide	Source	Catalogue No.
*Primary*					
Cav-1	Mouse	1:100	Yes	BD Transduction Labs.	610,406
nNOS-C	Guinea pig	1:100	No	Euro-Diagnostica	B-GP225-1
nNOS-C	Rabbit	1:100	No	Affinity Bioreagents	PA3-032A
HuC/D	Mouse	1:100	No	Molecular Probes	A21271
*Secondary*					
Cy3-conjugated donkey antimouse IgG				Jackson Immuno Research Labs.	715-165-151
FITC-conjugated donkey anti-guinea pig IgG				Research Diagnostics	706-095-148
*Sera*					
Normal donkey serum				Calbiochem	566,460

Cav-1, caveolin-1; nNOS-C, neuronal nitric oxide synthase, COOH- epitope; HuC/D, HuC/HuD neuronal protein (Human).

#### Confocal laser scanning microscopic study

The immunolabelled cryosections and whole mount preparations were examined by confocal laser scanning microscope and saved by LSM 5 Image (Laser Scanning Microscopy program from Carl Zeiss, Toronto, Canada). The images immunolabelled with Cy3 were captured by helium/neon laser (wavelength 543 nm laser line) with long path 590 filter, and images immunolabelled with FITC or Alexa488 from Invitrogen (Burlingon, Canada) conjugated with secondary antibody, donkey anti-rabbit IgG) were taken by Argon laser (wavelength 488 nm laser line) with band path 500–530 IR filter. The resolution of the all images obtained from the confocal microscope was originally 512 × 512 pixels. In the obtained cryosection images mucosa and submucosa were eliminated, and the muscularis was extracted. In the whole mount preparation images longitudinal muscle layer was eliminated and the myenteric ganglia and plexus were extracted. All final images were enhanced by brightness, contrast, and gamma tool of LSM 5 image and edited by Adobe PhotoShop Version 7.0.

### Coimmunoprecipitation and Western blotting

Full-length small intestinal tissue was cleaned from any adhering mesenteric blood vessels and tissues. The intestine was opened on the mesenteric border and the mucosa was removed by scraping with a scalpel blade. All tissue manipulations were done in ice-cold Krebs-Ringer solution. The tissue was immediately frozen in liquid nitrogen and stored at -80°C till used. On the day of the experiment, frozen tissues were crushed under liquid nitrogen and put in a 50 mM Tris buffer (pH 7.4) containing 3.1 mM sucrose, 1 mM dithiothreitol, 0.1% Triton X-100 and 1:1000 protease inhibitor cocktail. The crushed tissues were homogenized on ice with a Polytron using three 20 s strokes separated by 1 min. cooling periods. Following homogenization, the crude homogenate was centrifuged at 12,000 ×*g* to remove large debris. The residual homogenate was divided into 500 μl aliquots. In some aliquots containing the calcium concentration was adjusted to 2 mM and calmodulin was added so that the final concentration was 5 μM. The aliquots were incubated with 5 μg/ml mouse anti-Cav-1 or mouse IgG negative control overnight at 4°C. 50 μl aliquots of a 50% slurry of protein G-coated sepharose beads were incubated with the mixture overnight at 4°C. The beads were separated from the homogenate by centrifugation at 12,000 ×*g*. The beads were washed three times in a high stringency buffer containing 500 mM NaCl, 1% IGEPAL CA-630, 50 mM Tris pH8.0, and 1 mM phenylmethanesulfonylfluoride. This was followed by one wash in washing buffer (50 mM Tris, pH 8.0). The beads were then suspended in an elution buffer containing 1% sodium dodecyl sulphate, 100 mM dithio-threitol, 3%β-mercaptoethanol, 50 mM Tris pH 7.5. The mixture was heated at 95°C for 3 min. followed by centrifugation to separate the beads. 1 μl of 0.1% bromophenol blue was added to the supernatant which was loaded on a polyacrylamide gel and blotted as described previously [11] using a 1:1000 mouse anti-nNOS (primary antibody) concentration. We were unable to carry out reverse immunoprecipitation owing to interference from the IgG light chain, which is in the same molecular weight range the Cav-1 band. Several commercially available kits were tried to overcome this problem without success.

### Materials

LNNA, apamin, iberiotoxin, ODQ, cyclopiazonic acid, charbachol nicardipine, methyl betacyclodextrin, WSC, phenylmethanesulfonylfluoride and protease inhibitor cocktail were purchased from Sigma (Oakville, ON, Canada). TTX and ω-conotoxin GVIA were purchased from the Alomone Labs (Jerusalem, Israel). Monoclonal mouse anti-Cav-1 and anti-nNOS were purchased from BD Transduction Laboratories (Mississauga, ON, Canada). Mouse IgG control was purchased from Chemicon (Temecula, CA, USA) The anti-nNOS antibody was the same one previously used [5, 15, 16], produced by Euro-Diagnostica and sold by Cedarlane (Mississauga, Canada). Calmodulin was purchased from Biogenesis (Kingston, NH, USA). Apart from apamin, all the drugs used in functional experiments were dissolved in double distilled water.

Apamin was dissolved in 0.05 M acetic acid which, on its own, did not affect the activity of the tissue in the amount added (10 μl in a 10 ml organ bath).

## Results

### Immunohistochemical staining of cryosections and whole mount preparations

**Fig. 1:**
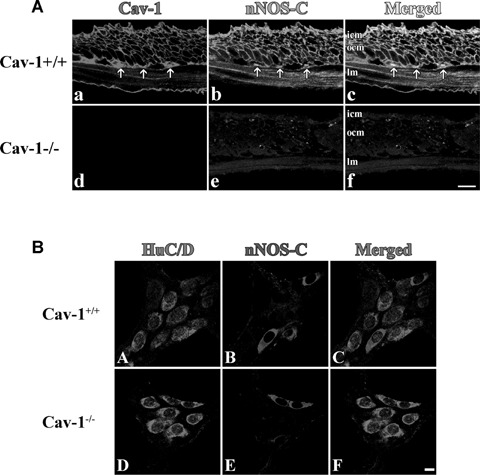
Immunostained cryosections (**A**) and whole mount preparations (**B**) of Cav1^+/+^ and Cav1^−/−^ mice small intestine. In section (**A**), panels (a-c) show caveolin-1 (a, red) and nNOS (b, green) immunoreactivity and their colocalization (c, yellow) in the smooth muscle cell membrane of Cav1^+/+^ mice small intestinal tissue. Panels (d–f) show the absence of both caveolin-1 (d) and nNOS (e) immunoreactivities in Cav1^−/−^ mice small intestine. Panel f is a merged image. The scale bar is 10 μm. icm, inner circular muscle layer; ocm, outer circular muscle layer and lm, longitudinal muscle layer. Arrows point to interstitial cells of Cajal in the myenteric plexus layer. Section (**B**) shows immunostained myenteric ganglia in Cav1^+/+^ (a–d) and Cav1^−/−^ (e–h) mice. Myenteric neuron cell bodies were localized by staining with HuC/D antibodies that stains the cytoplasm of neuron cell bodies (a and e, red). Panels b and e show neuron cells in myenteric ganglia stained with anti-nNOS COOH terminal epitope (blue). Panels c and f are merged images. Scale bars are 10 μm.

In experiments where primary or secondary antibodies were omitted, no immunoreactivity was seen. Similar to BALB/c mice [16], Cav1^+/+^ small intestine cryosections showed that Cav-1 and the putative nNOS immunoreactivity colocalized in smooth muscle plasma membrane (Fig. 1(A)a-c). The Cav1^−/−^ cryosections lacked immunoreactivity for both proteins (Fig. 1(A)d-f). On the other hand, examination of myen-teric ganglia in whole mount preparations from both Cav1^+/+^ and Cav1^−/−^ showed that nNOS is expressed in neuron cells of both mice strains (Fig.1B). An antibody to HuC/D protein was used to localize cell bodies of myenteric nerves (Fig. 1(B) a and e). Another antibody, made in rabbit against nNOS C-ter-minal epitope, which was previously reported to stain nNOS in the smooth muscle of the aorta of mice [23], also stained nNOS in the smooth muscle membrane of Cav1^+/+^ tissue. This staining was missing in the smooth muscle membrane in Cav1 ^−/−^ tissue (Data not shown). This antibody, however, was less specific for the membrane nNOS immunoreactivity and stained cytosolic sites in Cav1^+/+^ tissue and to a much lower extent in the Cav1^−/−^ tissue.

### Coimmunoprecipitation and Western blotting

**Fig. 2:**
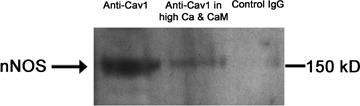
Representative blot of nNOS precipitated from intestinal homogenate with anti-caveolin-1 antibodies in control conditions and in presence of calmodulin and a high calcium concentration in addition to IgG negative control antibody. The amount of nNOS pulled down in high calcium and calmodulin is very much reduced. Control mouse IgG was used to show the selectivity of the coimmunoprecipitation. The blot is a representative of four experiments.

**Fig. 3:**
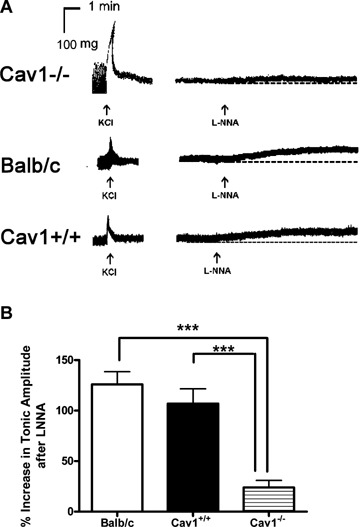
The effect of LNNA on contractile tone of intestinal tissue preparations following depolarization by KCl in BALB/c, Cav1^+/+^ and Cav1^−/−^ mice. (**A**) Representative tracings showing the increase in tone after LNNA treatment only in BALB/c and Cav1^+/+^ but not in Cav1^−/−^ mice. (**B**) The increase in contractile tone is much reduced in Cav1^−/−^ in comparison to the control mice. *n*-values are 12 for BALB/c, 6 for Cav1^+/+^ and nine for Cav1^−/−^. Results shown are the increase in tone normalized to the active tone before the addition of KCl. Statistical analysis was done by anova followed by Bonferroni *post-hoctest* and significance is denoted by **P< 0.01, and ***P< 0.001.

Immunoprecipitation of Cav-1 from BALB/c mouse small intestine homogenate followed by probing for nNOS showed an nNOS band at 150 kD (Fig. 3). Immunoprecipitation in the presence of high calcium and calmodulin greatly reduced the nNOS band intensity. Tissue homogenates were also immunoprecipitated with the control mouse IgG under the regular low calcium conditions. No nNOS band was detected in the lanes corresponding to these treatments (Fig. 2).

### Functional experiments

#### Spontaneous tone developed

The spontaneous tone developed in each of the tissues was measured (relative to tone in 0 calcium) before the addition of KCl and used as the 100% value to normalize the increase in tone that follows the addition of LNNA after KCl. The values of spontaneous tone developed were not different in BALB/c, Cav1^+/+^, and Cav1^−/−^ and were (in mg tension) 47.2 ± 6.3 (*n*= 12), 40.4 ± 6.3 (*n*= 6), and 45.7 ± 8.0 (*n*= 9), respectively.

#### Effect of LNNA on tissues contracted with KCl

Mouse small intestinal tissues responded to 60 mM KCl by a transient phasic contraction that gradually stabilized into a sustained increase in contractile tone (Fig. 3). Addition of 100 μM LNNA after the KCl contraction plateaued caused an increased in the tone of contraction in BALBalb/c and Cav1^+/+^ tissue segments (Fig. 3A). The increase in tonic contraction was very much reduced in the Cav1^−/−^ tissues when compared to the controls (Fig. 3).

#### Effect of cholesterol depletion on nNOS immunoreactivity and tissue response to LNNA

**Fig. 4:**
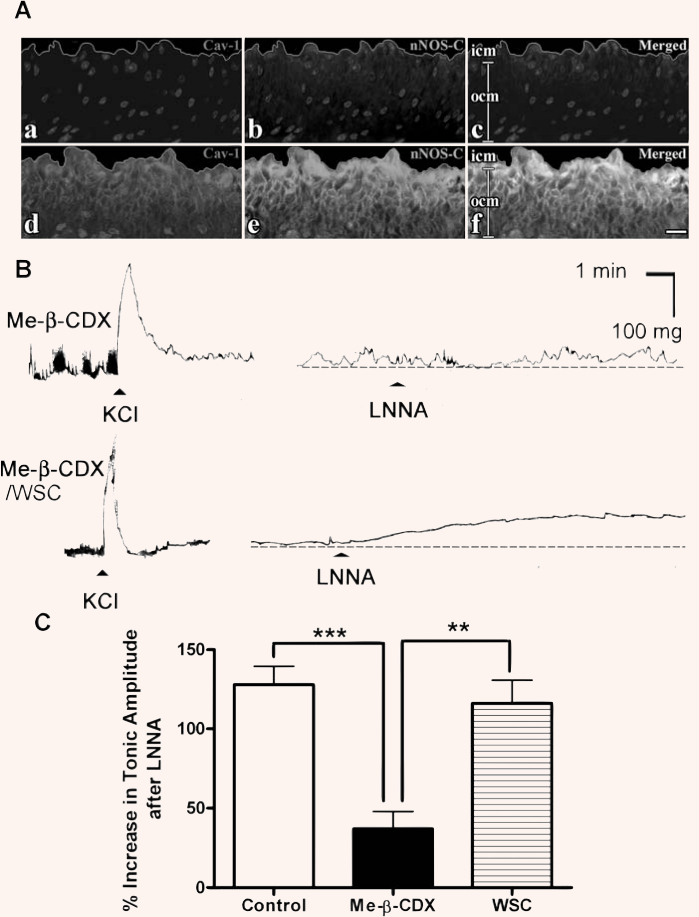
The effect of caveolae disruption by cholesterol depletion on the immunoreactivities of caveolin-1 and nNOS in smooth muscle cell membrane and the response to LNNA following tissue depolarization by KCl in BALB/c mouse small intestine. (**A**) immunostained cryosections of BALB/c mice small intestinal tissues showing the effects of Me-β-CDX treatment (a–c) and Me-β-CDX followed by WSC panels (d–f). caveolin-1 (a, red) and nNOS (b, green) immunoreactivities are very much reduced following Me-β-CDX treatment. The immunoreactivities are restored by WSC (d for caveolin-1, red and e for nNOS, green). Panels c and f are merged images showing the colocalization of caveolin-1 and nNOS in smooth muscle cell membrane (yellow) that is restored after WSC treatment. Scale bars are 10 μm. (**B**) Representative tracings showing that cholesterol depletion reduced the increase in tone by LNNA and that this was restored following treatment with WSC to replenish membrane cholesterol. (**C**) increase in tone after LNNA is added following KCl normalized to the active tome before addition of KCl. *n*-values are 13 for the control and five for Me-β-CDX and Me-β-CDX/WSC treated tissues. Statistical analysis was done by anova followed by Bonferroni post hoc test and significance is denoted by *P < 0.05, **P< 0.01, and ***P< 0.001.

Plasma membrane cholesterol was depleted by incubating BALB/c tissue preparations for 1 hr in 40 mM Me-β-CDX. In these tissues the immunoreactivities of both nNOS and Cav-1 in smooth muscle cell membrane were reduced (Fig. 4A). Although these tissues maintained their phasic activity and responded to KCl by contraction, they showed very little increase in contractile tone when LNNA was added following KCl (Representative tracing in Fig. 4B). However, when 2.54 mM WSC was used to replete the membrane cholesterol following depletion, the immunoreactivities of nNOS and Cav-1 in the smooth muscle plasma membrane were restored and the response to LNNA became similar to control levels (Fig. 4).

#### Effects of agents altering intracellular calcium levels

**Fig. 5:**
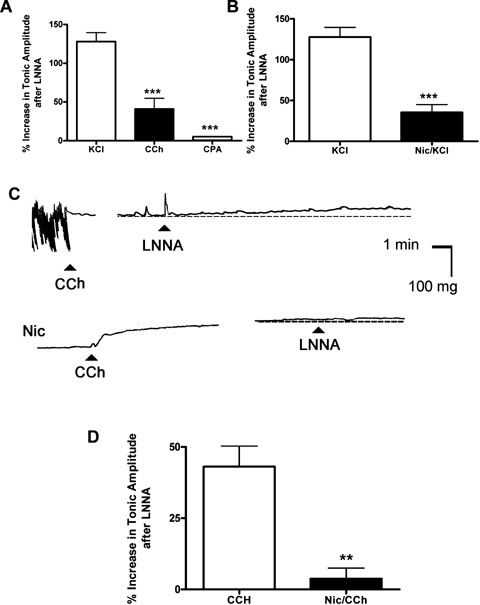
The increase in contractile tone by LNNA following different agents affecting intracellular calcium in BALB/c mice intestinal tissue. (**A**) The largest increase in contractile tone following LNNA was seen after tissue depolarization by KCl. Carbachol (CCH), which activates L-type calcium channels and releases calcium from intracellular stores, only showed a partial increase in tone when LNNA was added. Blocking the SERCA pump by cyclopiazonic acid did not cause an increase in the contractile tone when LNNA was added. *n* -values are 13 for KCl and 6 for CCH and cyclopiazonic acid. Statistical analysis was done by anova followed by Bonferroni *post-hoc test and* significant difference from KCl is denoted by ***P< 0.001. (**B**) Blocking the L-type calcium channels by nicardipine prior to tissue depolarization by KCl markedly reduced the increase in contractile tone when LNNA was added to the tissues. (**C**) Representative tracings showing the tissue response to CCh and the increase in tone after the addition of LNNA. Nicardipine-treated tissues still responded by contraction to CCh, however, they showed no response to LNNA afterwards. (**D**) Blocking the L-type calcium channels by nicardipine prior to the addition of CCH almost abolished the increase in contractile tone when LNNA was added to the tissues. *n*-values are 5 for the nicardipine treated tissues. Statistical analysis was done by t-test and significance is denoted by **P < 0.01 and ***P < 0.001.

The response to LNNA added after the tissue was incubated with compounds presumed to increase the intracellular cytosolic calcium level, but not primarily by opening L-type calcium channels, was studied in BALB/c mouse tissue. The sarco/endoplasmic reticulum Calcium ATPase (SERCA) inhibitor, cyclopiazonic acid (10 μM), was incubated with the tissue to block the reuptake of the cytosolic calcium into the sarcoplasmic reticulum. In other tissues 10 μM carbachol was used to increase intracellular calcium by release from the sarcoplasmic reticulum and activation of L-type calcium channels. Addition of 100 μM LNNA 5 min. after cyclopiazonic acid did not result in an increase in the tone of contraction while LNNA after carbachol resulted in a small, significant increase in contractile tone (Fig. 5A). On the other hand, pre-treat-ment of the tissues with the L-type calcium channel blocker, nicardipine (1 μM), during the equilibration period abolished the tissue response to KCl and greatly reduced the response to LNNA added afterwards (Fig. 5B). In addition, nicardipine only reduced the contractile response to carbachol, however, it abolished the response to LNNA added afterwards (Fig. 5C and D). Similar experiements with cyclopiazonic acid and carbachol in Cav1^−/−^ tissues showed no increase in the contractile tone when LNNA was added (Data not shown).

#### Effect of blocking nitric oxide action

**Fig. 6:**
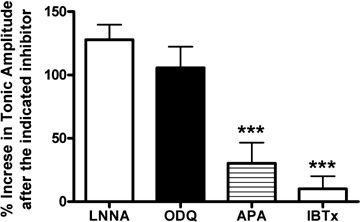
The effects of different agents acting on the signal transduction mechanisms downstream of nitric oxide on BALB/c intestinal tissue following depolarization by KCl. The increase in tone was achieved only by prevention of nitric oxide synthesis by LNNA or inhibition of soluble guanylate cyclase activity by ODQ. Blocking of small and large calcium-activated potassium channels did not produce an increase in contractile tone that is significantly different from zero. *n*-values are 13 for LNNA, 6 for ODQ and apamin and 5 for iberiotoxin. Statistical analysis was done by anova followed by Bonferoni *post-hoc test* and significant difference from KCl/LNNA is denoted by ***P< 0.001.

The effects of some pharmacological agents that block the action of nitric oxide on intracellular targets added after tissue depolarization with KCl were studied. 1 μM of the soluble guanylate cyclase inhibitor ODQ, added after the KCl contraction plateaued, increased the tone of the contraction similar to LNNA. However, small and large calcium-activated potassium channel blockers, apamin (1 μM) and iberiotoxin (0.1 μM), did not produce an increase in the contractile tone (Fig. 6).

## Discussion

In the present study we used immunohistochemistry and coim-munoprecipitation to show the association of Cav-1 and a putative nNOS variant in the small intestine of BALB/c mice. Previous studies showed that nNOS and Cav-1 can interact directly *in vitro*[9] or indirectly through binding to the dystrophin complex [24]. This complex interacts directly with the caveolin proteins [25]. Here we also showed that the interaction between Cav-1 and nNOS is weakened in the presence of calmodulin and a high-calcium concentration. This provides evidence in a native model that nNOS is regulated by Cav-1 in a manner similar to eNOS by interference with calcium and calmodulin binding.

On the other hand, in previous studies [18, 19], imunohisto-chemical examination showed that tissues from Cav1^−/−^ lacked, in addition to Cav-1, the putative nNOS variant expressed in the smooth muscle plasma membrane but not the nNOS expressed in the myenteric plexus nerves. This alteration further supports the association between Cav-1 and nNOS in smooth muscle plasma membrane in the small intestine. Moreover, since they only lack nNOS in smooth muscle cells but not myenteric neurons, Cav1^−/−^ mice offer a convenient model to act as a negative control in the study of the putative smooth muscle nNOS function.

Since the activity of nNOS is calcium dependent [26], we tested the function of the smooth muscle NOS using agents that increase intracellular calcium concentration. To avoid the interference from activation of nNOS present in enteric neurons, our experiments were conducted after blocking the activity of the enteric neurons using TTX and ω-conotoxin GVIA. In BALB/c and Cav1^+/+^ tissues, activating the smooth muscle L-type calcium channels by depolarization with 60 mM KCl caused a phasic contraction that stabilized into a sustained plateau phase. At this stage LNNA was added to block the smooth muscle nNOS activity. The resulted increase in the tone of contraction indicates that this enzyme was active in producing nitric oxide which modulated the smooth muscle contraction in response to KCl. On the other hand, Cav1^−/−^ tissues that lack the putative nNOS in smooth muscle, showed a very small increase in the tone of contraction when LNNA was added after KCl indicating that the modulatory role of nNOS on the contraction is absent.

In these experiments, we used Cav1^+/+^ controls in addition to BALB/c mice to show that the absence of the LNNA response in Cav1^−/−^ mice is linked to the absence of the putative nNOS from smooth muscle cells, rather than because of a strain variation. Cav1^−/−^ mice also lack the interaction between Cav-1 and eNOS and this was shown to lead to increased eNOS activation [27]. We considered a similar scenario in our model whereby nNOS gets separated from Cav-1 and assumes a cytosolic localization, though our immunohistochemistry results failed to locate it. A cytosolic location is also unlikely because, if cytosolic and not associated with caveolae, nNOS will be less prone to activation by calcium coming through L-type calcium channels and more prone to activation by calcium released from the intracellular stores. However, in our experiments with cyclopiazonic acid and carba-chol on Cav1^−/−^ tissues, LNNA showed no response. The fate of the nNOS missing from the plasma membrane in Cav-1 knockout intestine is unclear. One possible location of the absent myogenic nNOS, yet to be tested, is that nNOS remained in the Golgi or in SR when caveolin 1 was absent. In any case, we do not suggest that its expression is abolished in Cav-1 knockout animals.

Further evidence to the role of putative smooth muscle nNOS in the modulation of the KCl contraction was provided by membrane cholesterol depletion experiments using BALB/c mouse tissue. We previously showed that treatment of mouse small intestinal tissues with Me-β-CDX to deplete membrane cholesterol resulted in the loss of Cav-1 immunoreactivity from the cell membrane and the loss of caveolae structures as shown using electron microscopy [28]. In the present study, we showed that disruption of caveolae by membrane cholesterol depletion reduced both nNOS and Cav-1 immunoreactivity in the smooth muscle membrane. This was expected as we showed that nNOS is associated with Cav-1. Me-β-CDX-treated tissues maintained their phasic activity and responded normally to KCl. However, these tissues responded very weakly to the addition of LNNA after KCl. On the other hand, incubation of the Me-p-CDX-treated tissues with WSC to replete membrane cholesterol restored the response to LNNA and also the plasma membrane Cav-1 and nNOS immunoreactivities. These results provide further evidence to the proposed function of nNOS; that is., that it requires the presence of the putative nNOS in intact caveolae structures in the plasma membrane of smooth muscle.

**Fig. 7:**
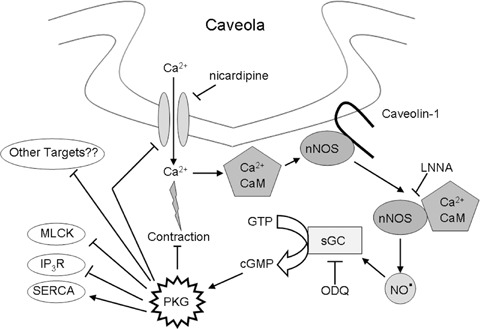
A demonstration of the hypothesized role of smooth muscle nNOS in the regulation of contraction induced by depolarization. Activation of L-type calcium channels and calcium influx triggers contraction as well as the activation of nNOS through calmodulin and dissociation form caveolin-1. The activated NOS produces nitric oxide that in turn activates soluble guanylate cyclase to produce cGMP which triggers a number of mechanisms to counteract the contraction. (CaM, Calmodulin; sGC, soluble guanlylate cyclase; PKG, protein kinase G; MLCK, myosin light chain kinase; IP3R, receptor for inositol triphosphate; SERCA, sarco(endo)plasmic reticulum Ca^2+^ ATPase; ODQ, 1H-[1,2,4]oxadiazolo[4,3-a]quinoxalin-1-one.

In addition to KCl, which activates L-type Ca^2+^ channels, we examined the effects of other agents which alter the level of cytosolic calcium. Addition of LNNA after the SERCA pump inhibitor, cyclopiazonic acid, which presumably increases cytosolic calcium levels by preventing the uptake of calcium into the sarcoplasmic reticulum, did not produce an increase in contractile tone. On the other hand, carbachol being a non-selective cholinergic agonist, is reported to induce smooth muscle contraction in mouse small intestine *via* two pathways [29]. First is the activation of M_3_ receptors and the release of intracellular calcium and then activation of M_2_ receptors which involves calcium entry through L-type calcium channels. The addition of LNNA to tissues treated with carbachol resulted in an increase in the tone of contraction that was less than in case of KCl stimulation. This agrees with the partial dependence of the contractile effect of carbachol on activation of L-type calcium channels in mouse small intestine. The importance of the activation of L-type calcium channels to obtain a response to LNNA was shown further by the absence of a response to LNNA after either KCl or carbachol in tissues pre-treated with the L-type calcium channel blocker, nicardipine. We previously showed [16] that, although L-type calcium channels are not closely colocalized with Cav-1 in mouse small intestine, they are associated with it. This association makes the increase in cytosolic calcium concentration due to L-type calcium channel activation higher in the caveolar domains very near to the smooth muscle nNOS (see Fig. 7). In contrast there may be a diffuse increase or accumulation of calcium brought about by the release of intracellular calcium or blockade of its uptake, even though caveolae are very close to peripheral SR at some sites and may provide a special space for calcium handling [30, 31], reviewed in [32].

Following NOS activation the biosynthesized nitric oxide relaxes (or reduces the contraction of) smooth muscle through a number of mechanisms. The main intracellular receptor for nitric oxide is soluble guanylate cyclase. Blocking soluble guanylate cyclase activity with ODQ following smooth muscle depolarization with KCl led to an increase in contractile tone that was similar to the use of LNNA. This indicates that nitric oxide produced by the smooth muscle nNOS activates soluble guanylate cyclase that produces cGMP. The latter in turn causes smooth muscle relaxation through different mechanisms [reviewed in 33]. The activation of calcium-activated potassium channels by nitric oxide in smooth muscle cells can occur independent of cGMP [34] leading to relaxation. In the present study we examined the role of both large conductance and small conductance calcium-activated potassium channels in the proposed action of the smooth muscle nNOS using the selective blockers iberiotoxin and apamin, respectively. However, neither agent had an effect on the contractile tone when added after KCl.

The present study did not examine the exact identity of the putative smooth muscle nNOS splice variant. This requires the isolation of pure preparations of small intestinal smooth muscle cells to act as a pure source of mRNA for this enzyme. That is beyond the scope of this study. However, the guinea pig antibody used in this study was raised against an epitope sequences selective to nNOS, and did not recognize eNOS in the endothelium of blood vessels (not shown).

The results of the present study suggest that the nNOS expressed in the mouse small intestinal smooth muscle is involved in the modulation of contraction resulting from L-type calcium channels activation by an nitric oxide and cGMP-dependent mechanism. L-type calcium channel activation is essential for the rhythmic contractions of smooth muscle in mouse small intestine that are initiated spontaneously by the interstitial cells of Cajal [35]. These spontaneously-paced rhythmic contractions of smooth muscle occurring in almost all mammalian guts are an important part of the normal physiological function of the intestine. Disruption of this function leads to serious effects on the overall intestinal function such as that observed in conditions of chronic idiopathic intestinal pseudo-obstruction [36]. However, the implications of the results of the present study on the pathophysiological changes in the intestine remain to be determined.
